# Biostimulatory activities of *Ascophyllum nodosum* extract in tomato and sweet pepper crops in a tropical environment

**DOI:** 10.1371/journal.pone.0216710

**Published:** 2019-05-14

**Authors:** Omar Ali, Adesh Ramsubhag, Jayaraj Jayaraman

**Affiliations:** Department of Life Sciences, Faculty of Science and Technology, The University of the West Indies, St. Augustine, Trinidad and Tobago; Zhejiang University, CHINA

## Abstract

This study evaluated the effectiveness of a commercially available *Ascophyllum nodosum* alkaline extract as a plant growth stimulant and defense elicitor against foliar diseases of tomato and sweet pepper caused by *Xanthomonas campestris* pv. *vesicatoria* and *Alternaria solani* in a tropical environment. Foliar applications of 0.5% *A*. *nodosum* extract (AN) at 10-day intervals resulted in significant (*P* < 0.05) increase in plant growth parameters, including plant height (40%), leaf number (50%), plant dry biomass (52%), root length (59%) and chlorophyll content (20%) compared to control. Treated plants also had a significantly higher number of flower clusters, flower numbers, fruits per cluster and total harvested fruit yield. The *Ascophyllum* extract significantly (*P* < 0.05) reduced disease incidence by the pathogens in both crops under greenhouse and field conditions. The combinatory treatment of seaweed extract and a minimum dose of contact fungicide in field trials, recorded the overall lowest disease levels (60% reduction) and highest yield (57% increase). Investigations into the mechanisms of disease suppression revealed the effects of the extract in inducing the activities of defense-related enzymes including phenylalanine ammonia lyase, peroxidase, polyphenol oxidase, chitinase and β-1,3-glucanase, as well as the levels of total phenolic compounds. The effect on SA, JA and ET-mediated signalling defense pathways was examined by quantifying expression levels of marker genes including *PR1-a*, *PinII* and *ETR-1*, for the above pathways respectively. Both crop plants treated with *A*. *nodosum* extract had significantly higher expression levels of the *PinII* and *ETR-1* marker genes than controls. This was coupled with a marked increase in gene transcripts involved in auxin (*IAA*), gibberellin (*Ga2Ox*) and cytokinin (*IPT*) biosynthesis, which provides possible evidence for induced growth in plants treated with AN extract. Cross-talks between growth and defense responses as a result of seaweed extract application could evidently implicate the benefits of seaweed extract usage in sustainable crop production.

## Introduction

Indiscriminate use of synthetic chemicals in agriculture has led to many challenges facing the sector globally, including development of pathogen and pest resistance, chemical residue carry-over in food produce, increased production cost and causation of irreparable negative impact on the environment [1]. Hence, there is great demand for developing and using novel alternative inputs in crop production. Within recent times, researchers have been exploring the use of organic-based biostimulants for enhancing plant growth and defense mechanisms [2]. One promising source of biostimulants is seaweed extracts which have been shown to possess both phytostimulatory and phytoelicitor properties [3,4]. Although the potential of extracts of several marine algal species has been demonstrated as plant biostimulants, the most important species used globally for commercial extraction is the brown seaweed *Ascophyllum nodosum* [5,6].

Plants possess the ability to shield themselves from a wide array of pathogenic attacks by regulating various inducible defense reactions. These reactions, however, involve the action of signalling molecules which arise from pathogenic stimuli or plant exposure to an external physical or chemical stress factor. Treatment of plants with seaweed extracts containing elicitor molecules can potentiate various inducible defence reactions. Induced resistance follows a sequence of activities which involve elicitor binding to specific receptor sites on the plant’s membrane. Following binding, the action of secondary chemical messengers further amplifies a signal which leads to downstream defense processes [6]. This activates a cascade of chemical reactions in the plant which contributes to an increase in resistance to invading pathogens. This broad-spectrum resistance allows the plant to protect itself against a wide array of pathogens and pests, including; bacteria, fungi, parasites, viruses, nematodes and insects [6]. The chemical stimulation which leads to induced resistance involves the phenylpropanoid pathway, the assembly of defense signalling molecules and even the build-up of antimicrobials encompassing pathogenesis-related (PR) proteins and phytoalexins [3,6]. These stimuli or plant elicitors which lead to the cascade of reactions, span an assorted group of compounds including proteins, glycoproteins, peptides, polysaccharides, oligosaccharides and lipids [7] and are commonly found in seaweed extracts [8]. Generally, these elicitors boost non-host plant resistance by mirroring the action of pathogens which activates self-protection of plants [9]. The actions of elicitors are facilitated by defense signalling molecules including salicylic acid (SA), jasmonic acid (JA) and ethylene (ET) which can lead to a systemic acquired resistance (SAR) or an induced systemic resistance (ISR) reaction [10,11,12]. Elicitor—signal transduction can therefore be a multiple network system which leads to an effective defense system by upregulating several defense reactions involving cross-linking of different signalling pathways capable of contributing different target responses [1,9,13].

Extracts prepared from seaweeds possess high levels of bioactive compounds which can beneficially prime the plant by affecting its metabolisms [13]. Seaweed extracts have been shown to contain a myriad of plant-bioactive organic and inorganic components including mannitol, laminarin, alginic acid, polysaccharides and oligosaccharides, vitamins, antioxidants, phytohormones (auxins, cytokinins, gibberellins and betaine) and low concentration of minerals (potassium, phosphorus, calcium, boron, magnesium, zinc and other trace elements) [7]. Therefore, apart from eliciting defense responses, the seaweed extracts can stimulate plant growth and enhance the photosynthetic rate [13,14]. Seaweed extracts, when utilized in cropping systems, have shown many benefits including, enhanced seed germination rates and seedling vigour, crop growth and yields, shelf life of produce, and significant decreases in disease damage caused by fungal, bacterial and viral pathogens [5,15].

Foliar and root drenching applications of *A*. *nodosum* extracts have been found to significantly reduce leaf and soil-borne diseases in carrot, cucumber and tomato [1,6,16]. This effect is credited to the components of the algal extract, most notably, polysaccharides such as laminarin and fucoidan, oligosachharides, carotenoids, betaines, osmoprotectants, cytokinins, sterols, amino acids, phenols and tannins which work synergistically to stimulate phytoelicitor and phytostimulatory responses in plants [17,18]. However, there is a dearth of data on the performance of *A*. *nodosum* extracts on crops grown in the tropical zone including the Caribbean region.

In the present study, a commercial alkaline extract of *A*. *nodosum* was evaluated for its comparative elicitor and phytostimulatory activity in tomato and sweet pepper crop plants under greenhouse and field conditions in the tropical island of Trinidad, Trinidad and Tobago, West Indies. The bioactivity of seaweed extract on disease suppression (bacterial spot caused by *Xanthomonas campestris pv vesicatoria* and early blight caused by *Alternaria solani*) was assessed. The above diseases are very serious in both crops in the Caribbean region and worldwide, which warrants urgent development of integrated management systems. Furthermore, due to the indiscriminate usage of fungicides, plant pathogens have been developing resistance to chemical pesticides [5]. Therefore, it is important for this region to develop and implement new disease control measures for both crops, that rely less on chemical-based strategies. Considering this problem, we investigated the use of a commercial Stimplex, *A*. *nodosum* seaweed extract as an alternative to chemical inputs. Furthermore, we have studied the mode of action of seaweed extract by assessing the induction of host defense mechanisms including defense enzyme activities and upregulation of defense-gene transcripts. Apart from the elicitor activities, the effects on crop growth and yield was also investigated at both wet and dry cropping seasons.

## Materials and methods

### Seaweed extract

The commercial seaweed extract product Stimplex (protein/amino acids 3–6%, lipid 1%, alginic acid 12–18%, fucose-containing polymers 12–15%, mannitol 5–6%, other carbohydrates 10–20%) prepared from *A*. *nodosum* (AN) was supplied by Acadian Seaplants, Limited, Dartmouth, NS, Canada. The *A*. *nodosum* extract was diluted to 0.5% concentration [1,5,16] in water and was used in all the experiments.

### Plant pathogen isolates

Virulent isolates of *Xanthomonas campestris* pv. *vesicatoria* (causal agent of bacterial spot) and *Alternaria solani* (causal agent of early blight) isolated from infected tomato leaf specimens were used in the inoculation studies.

### *In vitro* microbial activity of *A*. *nodosum* extract to pathogens

A disc diffusion assay was carried out to assess the antibacterial effects of the seaweed extract. A single colony of *X*. *c*. pv. *vesicatoria* was transferred to 9 ml of Mueller Hilton (MH) broth (Oxoid, England) which was incubated at 35°C in a shaking incubator (150 rpm) for 48 hrs. Bacterial concentration was adjusted to ~1.5 × 10^8^ CFU mL^−1^ using MacFarland standard and 100 μl of bacterial suspension was spread-plated onto the MH agar plates. Sterile filter paper discs (6 mm) impregnated with 50 μl of filter (0.45 μm)-sterilized 0.5% AN was placed onto the MH plates and incubated at 35°C for 48 h. The diameter of the zone of clearance around the discs was taken as a measure of bacterial inhibition. A copper-based fungicide (Cupraneb 2 g L^-1^) was used as the positive control. A hyphal growth inhibition assay was also performed to assess the antifungal activity of the seaweed extract on *A*. *solani*. Cores (5 mm) cut from the peripheral growth of a one-week-old plate culture of the fungus were placed onto fresh PDA and incubated for 24 h. Filter paper discs (6 mm) impregnated with 50 μl of sterile AN (0.5%) were then placed 3 cm away from the hyphal growth on the PDA plate and incubated at 25°C. Plates were observed for inhibition of hyphal growth and inhibition zones measured at the end of 72h. Cupraneb at 2 g L^-1^ was used as the positive control.

### Effect of *A*. *nodosum* extract on disease severity and plant growth under greenhouse conditions

Healthy 6-week old tomato seedlings and sweet pepper of the varieties Hybrid-61 and Amrit, respectively, were used in the study. The above varieties are commonly used by farmers in the southern Caribbean region. Seedlings were transplanted into pots (25 cm diameter) with a mix of peat moss and garden soil in a 1:1 ratio and drip irrigated. The plants were grown in a greenhouse at 25–30°C, 70–85% relative humidity and 600–1,000 μmol photons/m^2^ /s with ~12h photoperiod. Plants were fertilized using 20:20:20 N:P:K water soluble fertilizer as per the standard recommendation given to farmers by the Government of Trinidad and Tobago’s Ministry of Agriculture, Land and Fisheries (https://agriculture.gov.tt/category/publications/manuals/). After 20 days of transplanting, the plants were treated with the AN as a 0.5% foliar spray until run-off. Negative control plants were sprayed with water. Fungicides (Daconex—Chlorothalonil 1.2gL^-1^ or Cupraneb—Copper oxychloride 30% + Zineb 10% + Maneb 10% 2 g L^-1^) were used as positive controls. Treatments were arranged in a completely randomized design and a buffer barrier was laid in place between treatments to avoid cross over. Six hours after treatment application, plants were inoculated with a bacterial cell suspension of *X*. *campestris pv*. *vesicatoria* (1.5 x 10 ^8^ CFU mL^-1^) or a conidial suspension of *A*. *solani* (1 x 10 ^6^ spores mL^-1^). After inoculation, plants were kept in a humid chamber for about 48 hours. Treatment sprays were continued every 10 days, until three applications were achieved. The disease scores of plants recorded seven days after each treatment application and a six-point rating scale (1 = 0%, 2 = 1–10%, 3 = 11–25%, 4 = 26–40%, 5 = 41–55% and 6 ≥ 56%) was used to determine disease severity and calculate the Percent Disease Index (PDI) [16]. The plants were maintained at 25–30°C, 70–85% relative humidity and 600–1,000 μmol photons/m2 /s with ~12h photoperiod.

After 45 days of planting, plant height, leaf number and dry biomass (leaflet, stem and root) readings were taken. Chlorophyll content of mature leaves at the fourth node was measured for each treatment plant using a chlorophyll meter (atLEAF+, FT Green LLC, Detroit, USA) and the readings were presented as a ratio similar to SPAD units [19]. Thirty plants were maintained per treatment and the experiment was replicated twice under similar conditions.

### Effect of *A*. *nodosum* extract on disease severity and plant growth under field conditions

Two field trials were conducted per crop in farmer’s fields in Valencia (10°38'57.5"N 61°11'18.9"W), Trinidad, during the dry (January-May) and wet season (June- December). The treatments were, *A*. *nodosum* extract (AN); Fungicide; Control and AN alternating with Fungicide. The AN treatment consisted of a 0.5% seaweed extract foliar spray while the fungicide treatment comprised a rotation between Daconex (1.2 g L^-1^) and Cupraneb (2 g L^-1^). The AN alternating with Fungicide integrated treatment consisted of a cycle of two applications of 0.5% foliar sprays of seaweed extract followed by one fungicide spray. The purpose of the integrated fungicide treatment was to reduce but not eliminate fungicide sprays from the treatment regime. Six-week-old sweet pepper (Amrit) and tomato (Hybrid-61) seedlings were transplanted 100 cm X 50 cm apart on ridges and cultivated using a staking system. The experimental plots were arranged in a completely randomized design with three replicates and 200 plants per replicate. Plants were grown as per the practices recommended by the Government of Trinidad and Tobago’s Ministry of Agriculture, Land and Fisheries (https://agriculture.gov.tt/category/publications/manuals/) for tillage of land, crop fertilization, weed and insect pest management. Treatments were applied every 10 days after transplanting. The percent disease severity was recorded every seven days following methods described previously. Plant height, root and shoot dry weight were also measured at the end of the experiment (90 days post- transplanting). Fruit yield for each crop was recorded after each harvest and total fresh fruit yield was calculated at the end of the experiment. Various plant reproductive parameters including number of bearing clusters, number of flowers per cluster and number of fruits per cluster were also recorded.

### Absolute quantification of pathogen infection in plant tissues

Healthy tomato and sweet pepper plants were arranged in the greenhouse and treated with *A*. *nodosum* extract and inoculated with plant pathogens as described for the Greenhouse experiment above. Three plants from each crop plant were sampled at time points of 0, 12, 24, 48, 96h over the period of the experiment. Total genomic DNA was isolated from leaf samples using SDS-CTAB [3]. Standard curves were generated from the DNA isolated from *in-vitro* cultures of pathogens including *X*. *campestris* pv. *vesicatoria* and *A*. *solani*, for the purposes of absolute quantification. The standard curve was plotted by placing cycle threshold value (Ct) against standard 10-fold DNA dilution concentrations. The PCR efficiency was also calculated to ensure it was within the acceptable range (90–100%). Quantitative Polymerase Chain Reaction (qPCR) reaction contained 2x SYBR Green qPCR Master Mix -High ROX (Bioland Scientific) with a primer concentration of 0.5 μM specific to each of the pathogens (S1 Table) and 10–100 ng of DNA. qPCR conditions for *A*. *solani* (Sp-sol-1658 primer) were 95°C for 10mins; 40 cycles of 15s at 95°C and 60s at 60°C; and melt curve for 15s at 60°C and 15s at 95°C. qPCR conditions for *X*. *campestris* pv. *vesicatoria* (XV1 primer) were 95°C for 5min; 40 cycles of 30s at 95°C and 60s at 57°C; and melt curve for 15s at 60°C and 15s at 95°C. The regression line from the standard curve was utilized to assess the pathogens’ DNA concentration from each of the crop plant at each time point.

### Effect of *A*. *nodosum* extract on defense pathway marker genes and hormonal biosynthesis genes—Transcription levels

Real-Time Quantitative Reverse Transcription PCR (qRT-PCR) was employed to assess the expression of marker gene transcripts involved in the salicylic acid (SA), jasmonate (JA) and ethylene (ET) mediated defense pathways in both tomato and sweet pepper. qRT-PCR was also used to deduce the effects of AN on genes involved in biosynthesis of auxin, cytokinin and gibberellin. Healthy 4-week-old tomato and sweet pepper seedlings were sprayed with 0.5% of seaweed extract or water. Triplicate plant samples were collected at 0, 12, 24, 48, 72 and 96 h after foliar treatment. The leaf samples were frozen in liquid nitrogen immediately after collection and then stored at -80°C until use. Total RNAs were extracted from 500mg leaf tissue using Trizol reagent (Invitrogen). RNA was quantified using Jenway Genova NanoSpec and quality verified through gel run. Reverse transcription was carried out using the 5X All-In-One 1^st^ Strand cDNA Synthesis kit containing PowerScript Reverse Transcriptase (RTase) (Bioland Scientific), utilizing 1μg of RNA employing random primers in a 20μl reaction. cDNA samples were then quantified by real-time PCR using specific primers in an Applied Biosystems 7500 Fast Real Time PCR system (Life Technologies Corp.) and the data analysed using the 2^(-ΔΔCt^) method [3,5]. qPCR reaction was performed in a total of 20μl volume, using 1μl cDNA, 1μl forward primer, 1μl reverse primer, 10μl of 2x SYBR Green qPCR Master Mix -High ROX (Bioland Scientific LLC) and 7μl water. Each sample was run in triplicate along with a no-template cDNA control. Primer sequence details of genes are provided in S1 Table. The house-keeping gene, β- actin was employed as internal control for relative quantification [1,3,6]. Melt curve assays and a visualized single PCR product via gel electrophoresis was used to assess primer specificity. Standard curves were also done for all primer sets to ensure 95–100% efficiency and to assess detection limits. The experiment was repeated once under identical conditions.

### Assessment of defense enzyme activities and quantification of total phenols

To assess defense enzyme activities and quantify the levels of total phenols as influenced by application of AN, healthy four-week-old tomato and sweet pepper seedlings were foliar sprayed with 0.5% of each seaweed extracts and water control. Triplicate plants were collected at 0, 12, 24, 48, 72 and 96 h after foliar treatment application. The leaf samples were frozen in liquid nitrogen immediately after collection and then stored at -80°C until use. A crude protein extraction was done on the plant samples [3] which were then used for quantification of defense-related enzymes activity including phenylalanine ammonia lyase (PAL), peroxidase (POD), polyphenol oxidase (PPO), chitinase (CHI) and β-1,3 glucanase (GLU).

PAL activity was estimated by measuring changes in absorbance at 290 nm from the conversion of L- phenylalanine to trans-cinnamic acid. The amount of trans-cinnamic acid synthesized was calculated using its extinction coefficient of 9630 M^−1^ cm^-1^. Enzyme activity was expressed in fresh weight basis as nmol trans-cinnamic acid mg^−1^ min^−1^ of sample [3]. POD activity was estimated by measuring changes in absorbance, using pyrogallol as substrate. The results were expressed as change in absorbance at 420 nm mg^−1^ min^−1^ of protein [20]. PPO activity was estimated by measured changes in absorbance using catechol as substrate; 200 μl of 0.01 M was added to the reaction mixture to initiate the reaction. PPO activity was expressed as change in absorbance mg^-1^min^-1^ of protein [6]. Chitinase activity was estimated by quantifying the rate of N-acetylglucosamine production utilizing chitin from crab shells as a substrate. Chitinase activity was quantified by changes in absorbance at 585 nm and expressed as nmoles GlcNAc equivalents min^-1^ g^-1^ fresh weight [3]. β-1,3-glucanase activity was estimated as change in absorbance at 500nm using laminarin as substrate. The enzyme activity was expressed as μmols glucose released mg^−1^ min^−1^ of sample [3].

The total phenolic content was quantified using Folin-ciocalteu reagent, measured as change in absorbance at 750nm and expressed as μg catechol equivalents g^−1^ fresh tissue [6].

### Data analysis

All datasets were analysed utilizing the IBM SPSS Statistical software package, Version 23. The data was analysed for significant differences among treatments using analysis of variance (ANOVA) for datasets with three or more treatment groups and Student’s t-test where there were only two treatment groups. The significance (*P* < 0.05) amongst means were determined by Fisher’s protected LSD. Repeated measures ANOVA was done on the data for experiments that were done over different time intervals. Each time point was also analysed separately for significance using the Student’s t-test.

## Results

### In vitro antimicrobial activity of *A*. *nodosum* extract against plant pathogens

*In-vitro* antimicrobial assays were carried out to test the direct antimicrobial effects of the seaweed extract. AN showed no inhibition zones at 0.5% concentration for both pathogens tested. The positive control (fungicide) showed zones of inhibition of 12.8 ± 0.55 mm and 10.1 ± 0.23 mm for *A*. *solani* and *X*. *campestris* pv. *vesicatoria* respectively. This experiment clearly demonstrated the lack of antibacterial and antifungal activity of AN at the selected concentration to the fungal and bacterial plant pathogens studied.

### Effect of *A*. *nodosum* extract on plant disease severity and plant growth under greenhouse conditions

**Fig 1:**
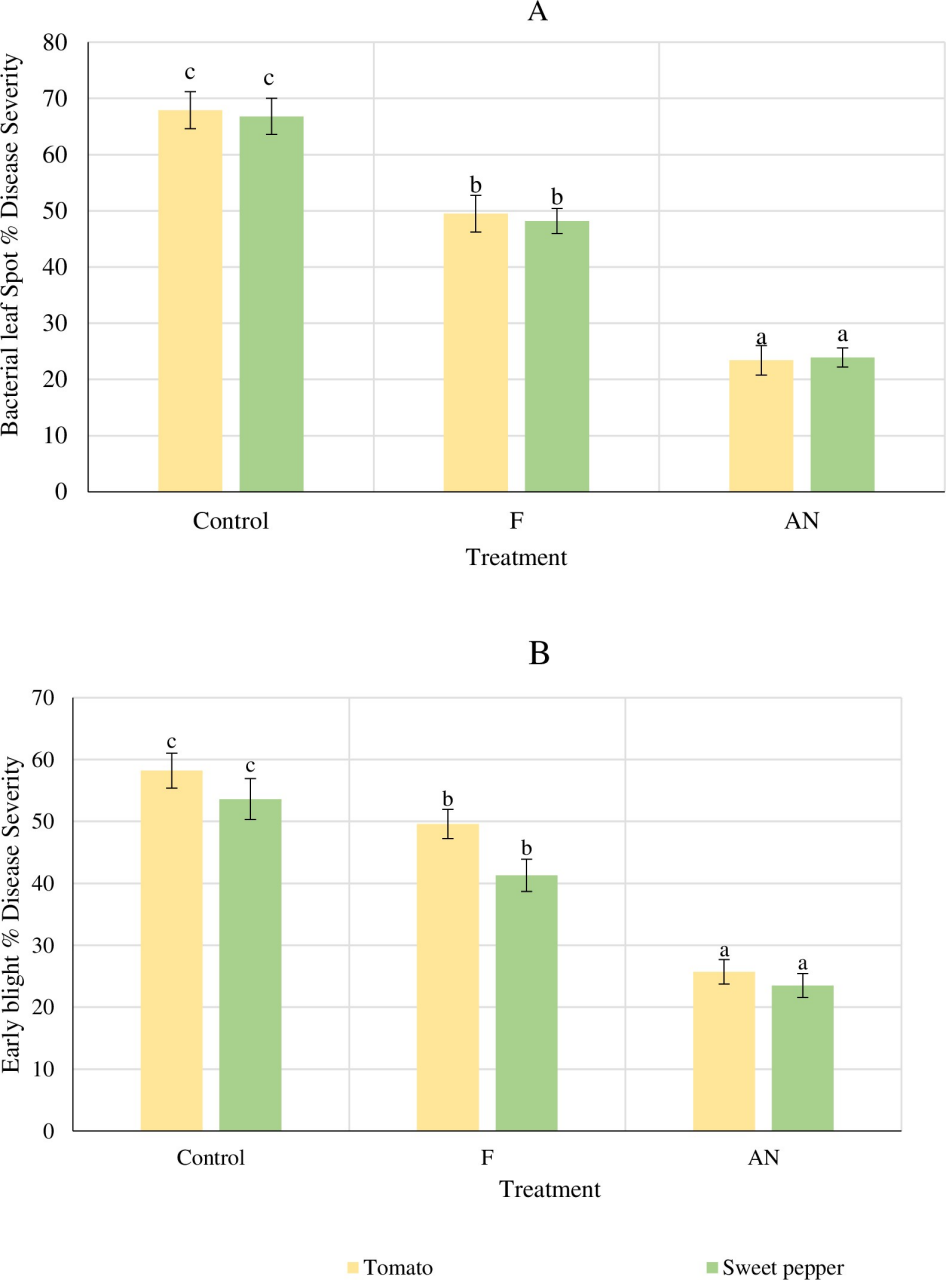
Effect *A*. *nodosum* extract on disease severity on greenhouse tomato and sweet pepper plants. **A: *Xanthomonas campestris* pv. *vesicatoria*; B: *A*. *solani*.** Treatments: AN- 0.5% foliar spray; F- Daconex—Chlorothalonil 1.2 g L^-1^ and Cupraneb; Copper oxychloride 30%+Zineb 10%+Maneb 10% 2 g L^-1^; C- Control. Values are means ± SD of % disease indices on 45 days after infection. n = 60 plants. Bars with different letters represents significant differences (*P* = 0.05) according to Fisher's least significant difference statistical test with vertical bars representing standard deviation. One-way ANOVA was used to compute statistical differences; Tomato (*A*. *solani*) = 1.0925 ^E-29^, Tomato (*X*. *campestris* pv. *vesicatoria*) = 1.126 ^E-40^; Sweet pepper (*A*. *solani*) = 8.535^E-20^ and Sweet pepper (*X*. *campestris* pv. *vesicatoria*) = 2.758^E-3^.

**Fig 2:**
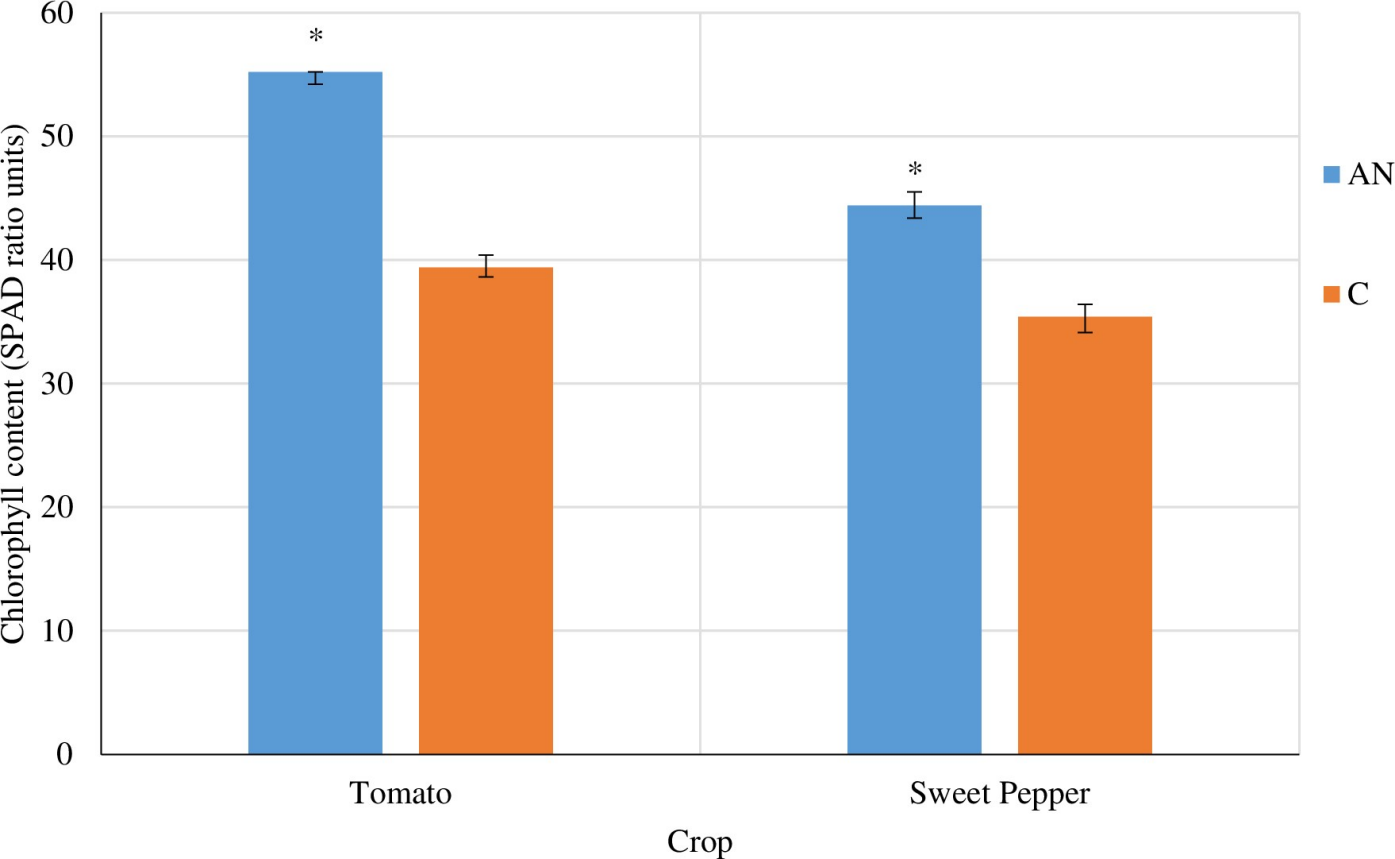
Effect of *A*. *nodosum* extract on chlorophyll content of greenhouse-grown tomato and sweet pepper. Treatments: AN- 0.5% foliar spray; C- Control. Values are means ±SD (n = 60). Student’s t-test (*P* -0.05) was used to compute significant differences among the extract and control on chlorophyll content 45days after treatment. * represents significant differences where p-value was less than 0.05.

The efficacy of AN to reduce disease levels and promote plant growth in tomato and sweet pepper was investigated. AN foliar spray was able to significantly (*P* < 0.05) reduce disease severity levels for both pathogens in tomato and sweet pepper (65.54%, 64.22% and 55.84%, 56.16% for *X*.*c*. pv *vesicatoria* and *A*. *solani*, respectively) compared to the control (Fig 1 and Table 1). Foliar application of AN significantly improved all the plant growth parameters including plant height, leaf number, root length and dry biomass compared to the fungicide (*P* < 0.05) (Tables 2 and 3). Additionally, leaf chlorophyll content was significantly greater in plants treated with AN (Fig 2) than control plants.

**Table 1:** Effect of *A*. *nodosum* extract on reduction of disease severity in greenhouse-grown tomato and sweet pepper.

Treatment	Tomato	Sweet pepper	Tomato	Sweet pepper
	Bacterial leaf spot (%) *		Early blight (%) *	
*A*. *nodosum*	65.54	64.22	55.84	56.16
Fungicide	27.09	27.84	14.78	22.95

*Percent disease reduction values—recorded 45 days after inoculation.

**Table 2:** Effect of *A*. *nodosum* extract application on plant growth parameters in tomato.

Treatments	Plant height (cm)	Leaf number	Root length (cm)	Total plant biomass (g)
*A*. *nodosum*	151.68 ±0.02 b	71.64 ±0.01 c	30.07 ±0.02 c	38.30 ±0.90 c
Fungicide	113.08 ±0.07 a	51.62 ±0.08 b	21.40 ±0.02 b	24.11 ±0.10 b
Water	102.56 ±0.10 a	34.80 ±0.12 a	14.44 ±0.03 a	17.24 ±0.10 a

Different letters denote significant differences at 95% confidence levels according to Fisher’s protected LSD.

**Table 3:** Effect of *A*. *nodosum* extract application on plant growth parameters in sweet pepper.

Treatments	Plant height (cm)	Leaf Number	Root length (cm)	Total plant biomass (g)
*A*. *nodosum*	90.54 ±0.03 c	30.62 ±0.08 c	25.14 ±0.20 c	22.50 ±0.04 c
Fungicide	63.70 ±0.23 b	19.52 ±0.10 b	15.04 ±0.10 b	13.60 ±0.44 b
Water	52.48 ±0.12 a	14.88 ±0.11 a	9.11 ±0.11 a	11.80 ±0.34 a

Different letters denote significant differences at 95% confidence levels according to Fisher’s protected LSD.

### Effect of *A*. *nodosum* extract application on plant disease severity and plant growth under field conditions

The bio-efficacy of AN in tomato and sweet pepper was investigated under farmer’s field conditions. Seaweed treatment alternating with fungicide significantly lowered disease severity levels compared to other treatments, both in the wet and dry seasons for both crop plants. *A*. *nodosum* extract was able to reduce disease severity by up to 50% compared to controls (Tables 4 and 5). Fruit yield was significantly higher for AN + fungicide treatment (up to 40%) than other treatments (Table 6). Additionally, both the seaweed extract and the combo treatment (AN + fungicide treatment) yielded higher numbers of flowers, fruits, flowers and fruits per cluster in tomato and sweet pepper (Table 7).

**Table 4:** Effect of *A*. *nodosum* extract application on disease severity of field-grown tomato and sweet pepper in the dry season.

Treatments	Dry Season			
	Tomato	Sweet pepper	Tomato	Sweet pepper
	Bacterial Spot (% PDI)		Early blight (%PDI)	
Control	77.16 ±1.22 d	71.02 ±1.88 d	70.10 ±1.22 d	69.08 ±1.70 d
F	49.41 ±1.83 c	48.21 ±1.99 c	50.90 ±1.55 c	47.12 ±1.22 c
AN	30.24 ±1.35 b	30.10 ±1.81 b	38.84 ±1.95 b	36.26 ±1.52 b
AN + F	19.94 ±1.89 a	23.15 ±1.09 a	30.07 ±1.57 a	27.95 ±1.79 a

Different letters denote significant differences at 95% confidence levels according to Fisher’s protected LSD.

**Table 5:** Effect of *A*. *nodosum* extract application on disease severity on field-grown tomato and sweet pepper in the wet season.

Treatment	Wet Season			
	Tomato	Sweet pepper	Tomato	Sweet pepper
	Bacterial Spot (% PDI)		Early blight (% PDI)	
Control	87.93 ±1.55 c	91.06 ±1.12 d	88.95 ±1.29 d	89.95 ±2.12 d
F	55.99 ±2.20 b	62.86 ±1.33 c	59.61 ±1.34 c	60.80 ±2.33 c
AN	48.99 ±1.40 b	44.98 ±1.23 b	51.91 ±1.33 b	51.74 ±1.34 b
AN + F	33.05 ±1.23 a	35.85 ±1.45 a	41.91 ±1.32 a	40.87 ±1.38 a

Different letters denote significant differences at 95% confidence levels according to Fisher’s protected LSD.

**Table 6:** Effect of *A*. *nodosum* extract application on fruit yield (kg) of field-grown tomato and sweet pepper in the dry season and the wet season.

Treatment	Dry Season		Wet Season	
	Tomato (kg)	Sweet Pepper (kg)	Tomato (kg)	Sweet Pepper (kg)
AN+F	358.14 ±1.33 d	454.93 ±1.01 d	286.84 ±1.01 d	302.27 ±1.75 d
AN	258.15 ±1.14 c	364.10 ±1.99 c	239.91 ±1.23 c	254.66 ±1.07 c
F	232.98 ±1.01 b	256.01 ±1.56 b	187.06 ±1.23 b	203.11 ±1.01 b
C	167.79 ±1.95 a	212.04 ±1.11 a	122.70 ±1.55 a	129.92 ±1.22 a

Different letters denote significant differences at 95% confidence levels according to Fisher’s protected LSD.

**Table 7:** Effect of *A*. *nodosum* extract application on plant yield parameters: Field-grown tomato (A) and sweet pepper (B).

	Number of bearing clusters		Number of flowers per cluster		Number of fruits per cluster	
	A	B	A	B	A	B
C	11.05 ±0.02 a	9.02 ±0.01 a	9.10 ±0.11 a	6.33 ±0.01 a	5.21 ±0.01 a	3.33 ±0.03 a
Fungicide	14.00 ±0.01 b	10.01 ±0.02 a	11.01 ±0.43 a	6.98 ±0.02 a	6.11 ±0.03 b	4.44 ±0.11 b
AN	19.00 ±0.01 c	13.44 ±0.02 b	17.87 ±0.02 b	10.99 ±0.04 b	9.88 ±0.11 c	6.98 ±0.01 c
AN+F	25.00 ±0.01 d	16.69 ±0.01 c	18.98 ±0.02 c	13.33 ±0.01 c	11.98 ±0.01 d	7.33 ±0.22 d

Different letters denote significant differences at 95% confidence levels according to Fisher’s protected LSD.

### Mechanism of induced resistance elicited by *A*. *nodosum* extract

#### Effect on colonization of pathogens in host tissues

**Fig 3:**
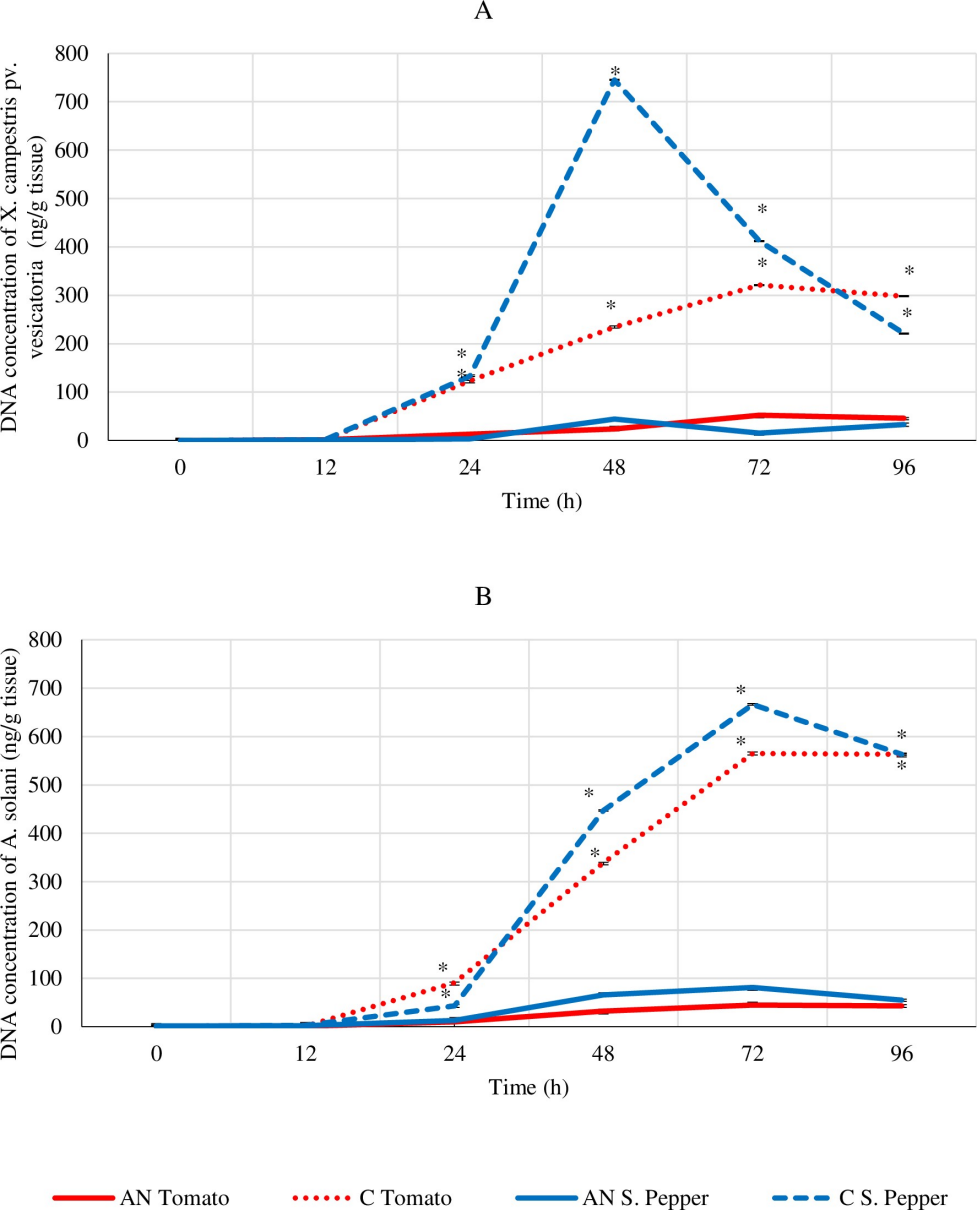
**Absolute quantification of A: *Xanthomonas campestris* pv. *vesicatoria*; B: *A*. *solani* genomic DNA in infected tomato and sweet pepper leaf tissue over time treated with *A*. *nodosum* extract.** The data represents the averages ±SD of 2 independent trials with 6 replicates. The data was analysed by repeated measures ANOVA to study the effects of time and treatment. The data was also analysed by Student’s t-test to examine the significance of seaweed extract versus control at each time point independently. * represents significant differences where p-value was less than 0.05.

Foliar treatment of AN was able to significantly (*P* < 0.05) reduce the load of both foliar pathogens, *A*. *solani* and *X*. *campestris* pv. *vesicatoria* in tomato and sweet pepper (Fig 3). Statistical analyses of each time point showed that times 24–96 h after exposure had significant reductions of both pathogens (*P* < 0.05).

#### Effect on activity of defence enzymes and total phenolic content

**Fig 4:**
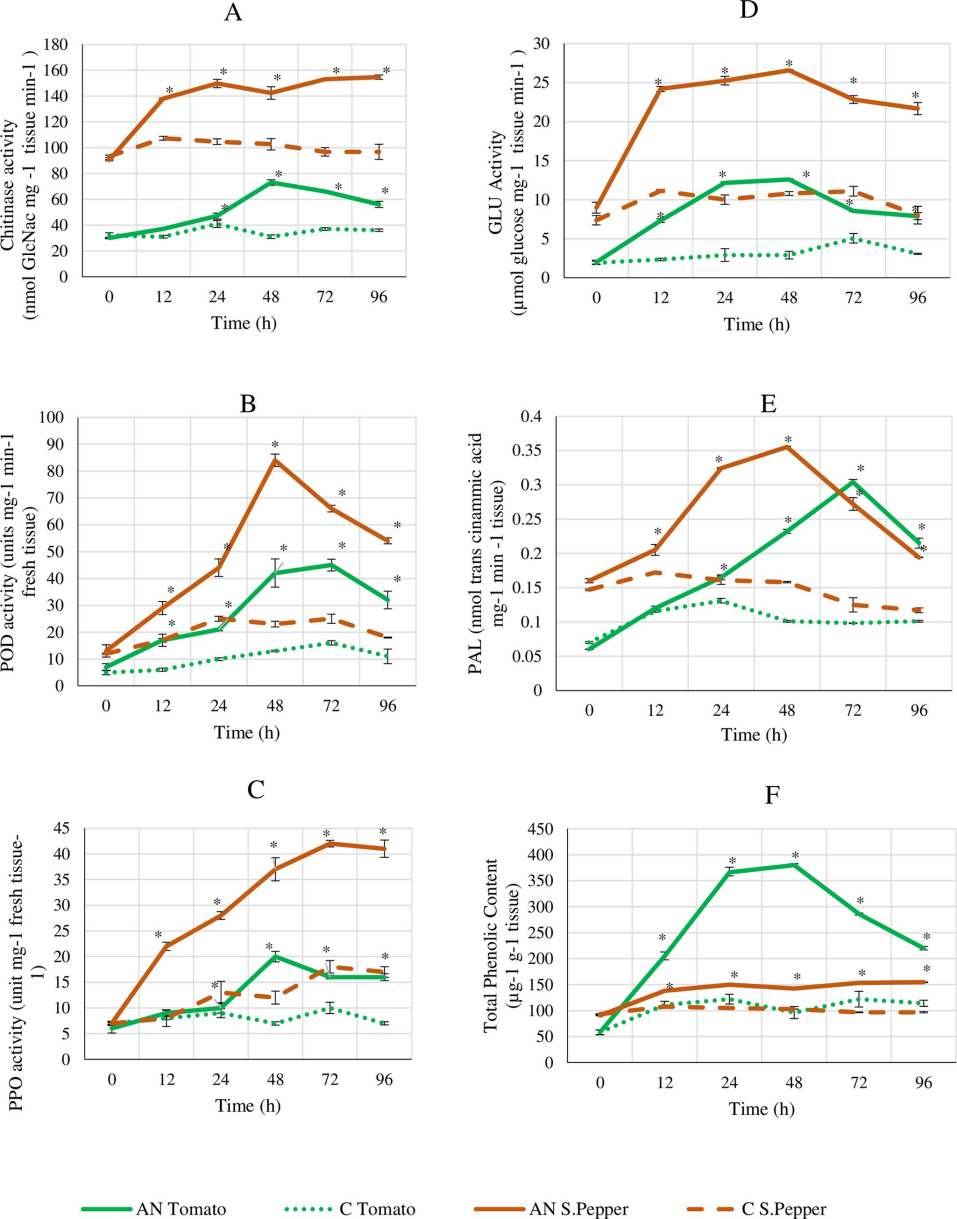
Activity of defense enzymes and quantitation of total phenolics in tomato and sweet pepper plants over time treated with *A*. *nodosum* extract. (A) Chitinase (CHI); (B) Peroxidase (POD); (C) Polyphenol oxidase (PPO); (D) Glucanase (GLU); (E) Phenylalanine ammonia lyase (PAL); (F) Total Phenolics. Leaf samples were collected at 0,12, 24, 48, 72 & 96h after seaweed extract application at 0.5% concentration. The data represents the averages ± SD of 2 independent trials with 6 replicates. The data was analysed by repeated measures ANOVA to study the effects of time and treatment. The data was also analysed by Student’s t-test to examine the significance of seaweed extract versus control at each time point independently. * represents significant differences where p-value was less than 0.05.

Tomato and sweet pepper plants were foliar treated with AN and the activities of defense enzymes were assayed and the total phenolic content quantified. Activities of defense enzymes including phenylalanine ammonia lyase (PAL), peroxidase (POD), polyphenol oxidase (PPO), chitinase (CHI) and β-1,3 glucanase (GLU) were assessed at 0, 12, 24, 48, 72 & 96 h after foliar treatment with AN. Repeated measures ANOVA analysis of results showed significant variation among the treatments on their effects. Student’s t-test showed significant (*P* < 0.05) increases in enzyme activity due to the application of AN extract as compared to the control at time points 12–96 h after treatment. PPO and POD peak activities were recorded at 72–96 h after treatment (Fig 4B and Fig 4C) while the activity of CHI, GLU and PAL were at peak at 24h after treatment (Fig 4A, Fig 4D and Fig 4E). Interestingly, similar trends were observed in both crop plants tested. Total phenolic levels were higher in all AN treated plants than the control, with peak levels at times 24-48h after treatment (Fig 4F). Total phenols in control plants did not change significantly over time, which established the baseline for the experiment (Fig 4F). Phenolic levels began to steadily decrease after 48h, then seemed to show constant levels up to 96h time point in both crop plants.

#### Effect on defense pathway marker gene expression

**Fig 5:**
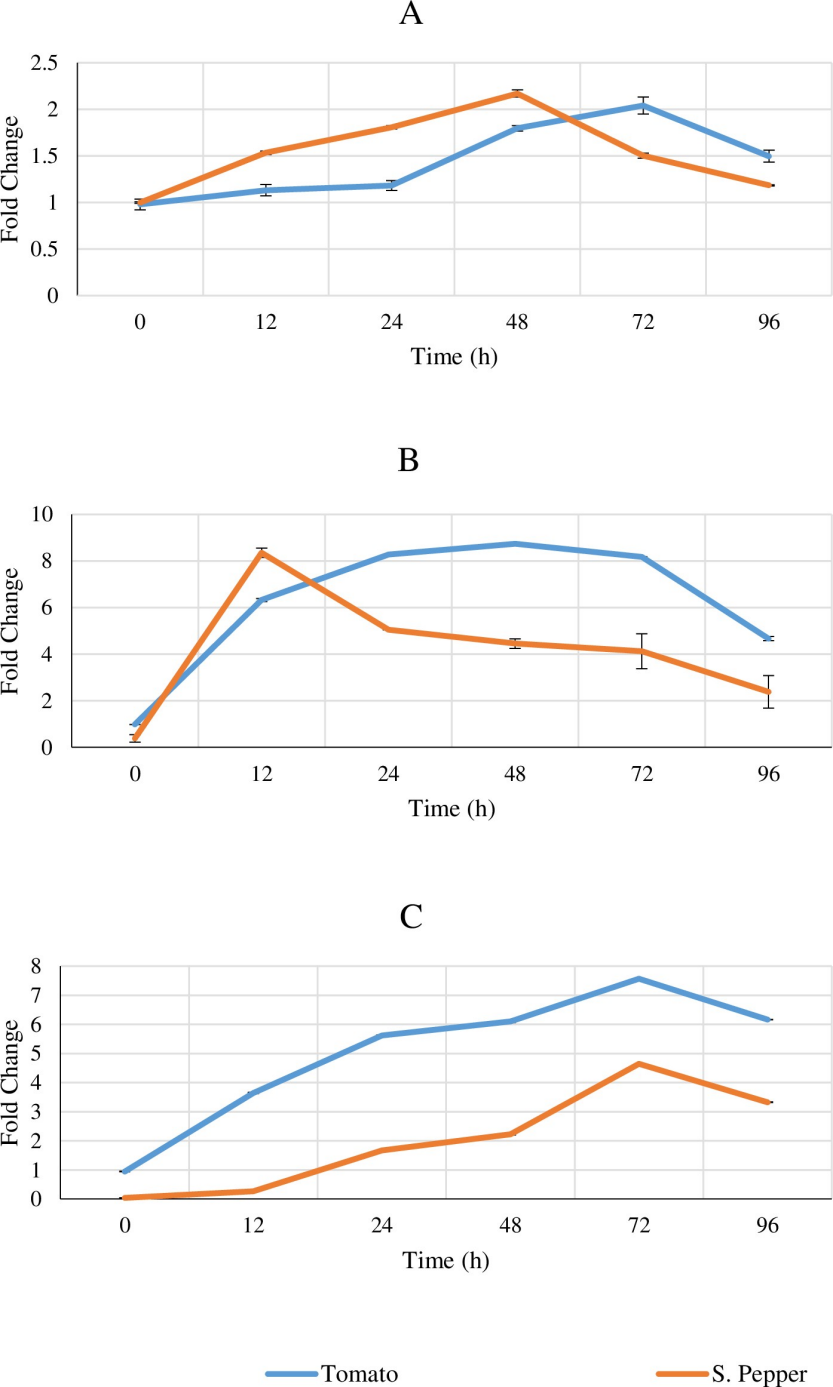
Real-time transcription of defense signalling pathway marker genes following *A*. *nodosum* extract application in tomato and sweet pepper plants. Plant tissue was collected at time points (0, 12, 24, 48, 72 & 96h) after seaweed extract application and relative qPCR done to monitor transcripts for (A) *PR-1a* (B) *PinII* (C) *Etr-1* genes. The data was normalized using the house keeping gene β-actin. Data represents fold change in comparison to the control. The values are averages ± SD for 2 independent trials with 6 replicates. The data was analysed by repeated measures ANOVA at 95% confidence interval to study the effects of time and treatment. The data was also analysed by Student’s t-test to examine the significance of seaweed extract versus control at each time point independently.

In an effort to understand the upregulation of defense marker gene expression upon AN application on tomato and sweet pepper, relative qPCR was performed using key marker genes involved in the SA, JA and ET pathways including *PR-1a*, *PIN II and ETR-1*. Repeated measures ANOVA showed that both tomato and sweet pepper plants treated with seaweed extract had significantly higher levels of *PIN II* and *ETR-1* gene transcripts than the control (*P* < 0.05). At time points 12–96 h after treatment, enzyme activity was higher (*P* < 0.05) in the seaweed extract treated plants based on Student’s t-test analysis. AN-treated plants showed early induction of *Pin-II* and *ETR-1* genes, with the peak rise at 12h and 24h in both crops (Fig 5B and 5C) and then a steady decline after 48h. However, there were no significant (*P* > 0.05) upregulation in the *PR-1a* gene transcripts for both crops treated with AN (Fig 5A).

#### Effect on hormonal biosynthesis gene transcript levels

**Fig 6:**
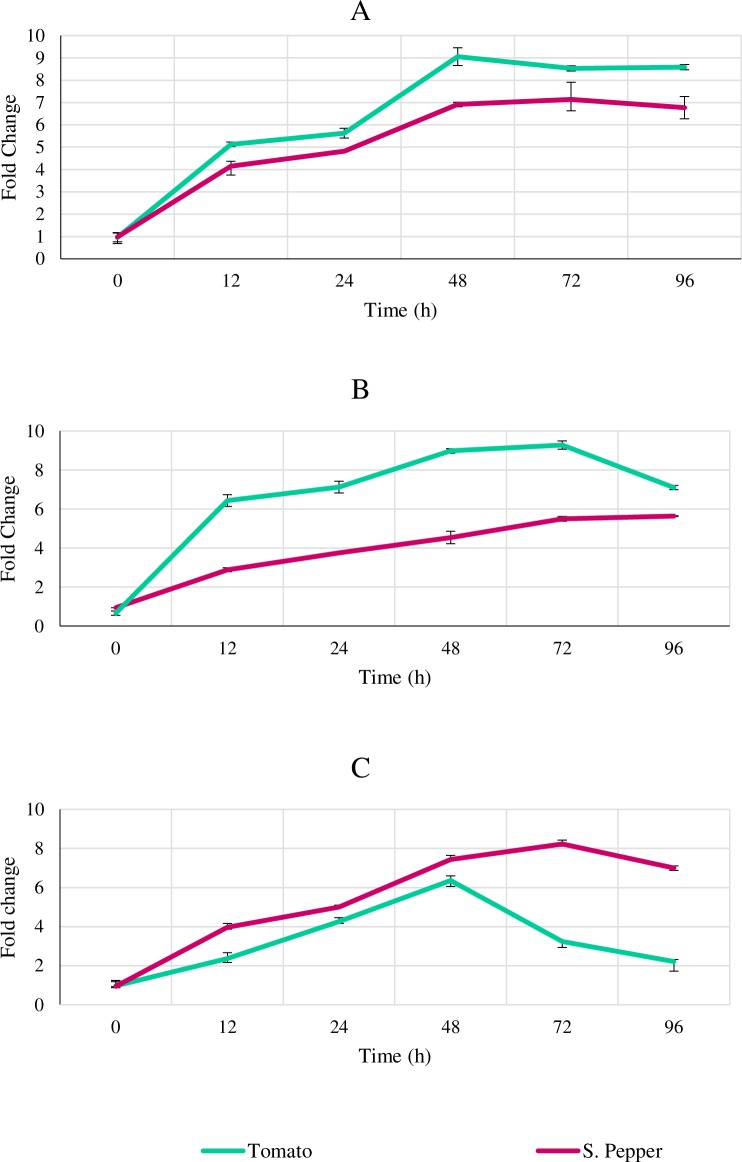
Real-time transcription of hormonal biosynthesis pathway genes following *A*. *nodosum* extract application in tomato and sweet pepper plants. Plant tissue was collected at time points (0, 12, 24, 48, 72 & 96h) after seaweed extract application and relative qPCR done to monitor transcripts for (A) *IPT* (B) *IAA* (C)*Ga2Ox2* genes. The data was normalized using the house keeping gene β-actin. Data represents fold change in comparison to the control. The data are averages ± SD for 2 independent trials with 6 replicates. The data was analysed by repeated measures ANOVA at 95% confidence interval to study the effects of time and treatment. The data was also analysed by Student’s t-test to examine the significance of seaweed extract versus control at each time point independently.

To deduce the probable mechanisms of induced plant growth implicated by seaweed extract treatment, transcription of hormonal biosynthesis genes including auxins, cytokinins and gibberellins was examined. Repeated measures ANOVA on the data showed that both tomato and sweet pepper plants treated with seaweed extract had significantly higher levels of gene transcripts (*IPT*, *IAA* and *Ga2Ox*) than the control (Fig 6A, Fig 6B and Fig 6C). At time points 12–96 h after treatment, the AN treated plant had statistically significant (*P* < 0.05) increases in enzyme activity as compared to the control, based on Student’s t-test analysis. All three gene transcripts showed highest upregulation and greatest fold change at 48–72 h after seaweed extract treatment.

## Discussion

The current study revealed the significant potential of *A*. *nodosum* extract to induce defense mechanisms and promote growth of tomato and sweet pepper plants in a tropical environment. *In vitro* antimicrobial tests of *A*. *nodosum* extract against the plant pathogens, *A*. *solani* and *X*. *campestris pv*. *vesicatoria* have demonstrated no direct antimicrobial effect at 0.5% concentration. However, both tomato and sweet pepper plants treated with the seaweed extract at 0.5% showed reduced disease severity levels of bacterial spot and early blight. The disease severity was even significantly less than the fungicide treatment. These trends were observed in both greenhouse and field trials conducted. Additionally, plants treated with the seaweed extract alternating with fungicide had the lowest levels of disease as well as the highest overall fruit yield. This is possibly due to the complementary action of both AN and fungicide where AN stimulates plant growth and plant defense responses and the contact fungicide works as a direct antimicrobial agent which tends to persist on the leaves of the plant preventing severe infections and secondary spread of the diseases [5,6]. All plant growth parameters measured also increased with the AN treatment compared to the controls for both tomato and sweet pepper. The induction of plant defense enzyme activities and elevation of total phenols as well as upregulation of defense marker genes were sustainably high in tomato and sweet pepper plants treated with AN. This induction of defense mechanisms may have led to the plants having lower disease levels compared to the controls.

Reduced infection levels in plants by application of the seaweed extract was further confirmed by lower levels of pathogen’s DNA via absolute quantitative PCR assays. This could be explained by the conditioning effect of these extracts on the plant system which caused induced resistance as opposed to having direct antimicrobial effects. This elicitor effect was elucidated through the enhancement of various plant defense enzymes coupled with the upregulation of defense marker gene transcripts. It has been theorized that various molecules present in *A*. *nodosum* extract can act as elicitors resulting in the stimulation of defense responses within the plant system which can counter against the pathogenic threat [6]. Increased levels of in-planta salicylic acid are associated with systemic acquired resistance (SAR) and expression of the *PR1-a* gene is linked to increased resistance to a wide variety of pathogens [3,11]. The marker genes of pathways involved in induced systemic resistance include the *Pin-II* (proteinase inhibitor) and *Etr-1* (ethylene receptor) genes. It is well documented that ethylene and jasmonates trigger an ISR response [16,21]. Plants treated with AN did not show any upregulation of the *PR-1a* gene in contrast to strong elicitation of the *Pin-II* and *Etr-1* genes. This demonstrated that both tomato and sweet pepper plants treated with AN had no activation of the SA-dependent mediated pathway but instead triggered JA and ET mediated signalling, which offered protection against the pathogens. Studies show that brown seaweed extracts contain laminarins, a β-1,3 glucan which negatively affects SA accumulation [17,22]. Laminarins present in these brown extracts accounts for about 30% of the entire composition and it elicits defense responses by mirroring the cell wall properties of various bacterial and fungal pathogens [22], thereby priming the defense systems in plants [3,6]. Reports have shown the elicitation of various plant defense enzymes and phenolics by seaweed extracts and it is well known that the ISR and SAR trigger increases in the activity of these enzymes [22,23]. Additionally, the increase in the activity of GLU and CHI enzymes are important since both are classed at pathogenesis-related proteins which play significant protective role during pathogenesis [6,16]. These enzymes hydrolyse both glucan and chitin which are polymers constituting the cell walls of many pathogenic fungi and were induced in plants when treated with the *A*. *nodosum* extract and *Gelidium serrulatum* [3]. Most studies have pointed to oligosaccharides and polysaccharides, such as laminarins and fucoidan, as being responsible for biostimulatory properties of seaweed extracts [3,24]. However, it is possible that other components present in the extracts including betaines and various phytohormones could contribute significantly to the well-being of the plant system. These could work together in an organized and harmonious manner to promote a healthy plant growth and productivity.

Phytostimulatory activities of *A*. *nodosum* extract were evident throughout the experiments as were evident from significant increases in plant growth parameters including plant height, root length, leaf number and total plant biomass. The results of the current study are also in agreement with reports on other crops including carrot, cucumber and tomato [1,6,16]. Additionally, the growth promoting effects of *A*. *nodosu*m has also been noted in crops grown in other tropical regions, including watermelon and cucumber [1,5]. The increased growth was much higher than those treated with seaweed extracts in a temperate environment [1,5] as well as in a subtropical region [3]. The increased efficacy of the extracts in tropical environments might be associated with enhanced availability of the active ingredients of the extract and increased responsiveness of the plant to the components of the extract. The effects of moderate stress in the tropical environment including water deficit during dry weather conditions and high light intensity might have been mitigated by the seaweed extract, which led to improved plant responses and performance in the field. The growth promoting properties observed may be as a result of the effects of phytohormones and growth regulatory substances present in the seaweed extracts and induced the biosynthesis of hormones by treated plants [1,3,6]. Indole acetic acid (IAA) and several cytokinins have been reported to have great growth promoting effects on plants [25,26]. IAA is responsible for a wide range of growth processes such as cell division, vascular growth, elongation and differentiation of roots and apical dominance. Cytokinins account for root: shoot ratio, nutrient mobilization and delay in senescence [27]. Cytokinins in the vegetative sections of the plant are linked to the partitioning of nutrients whereas high amounts of cytokinins in the reproductive organs are associated with the mobilization of nutrients [28]. Increased levels of cytokinin and mobilization of nutrients in vegetative and reproductive sections of plants have been reported to be influenced by seaweed extracts [1,17]. Plants treated with these extracts also showed higher levels of cytokinins in their roots which could possibly address the demand for cytokinins in the developing plant [3]. Upon application of seaweed extracts during the vegetative stage, tomato plants showed an increase in fresh fruit weight and fruit quality, which was attributed to the likelihood of the seaweed extracts promoting nutrient uptake which led to improved growth and vigour [14]. Seaweed extract also improved chlorophyll content in tomato and sweet pepper in this study, which was also supported by other reports [5,29]. This increase may be as a result of reduced degradation of chlorophyll due to the presence of betaines in the seaweed extract [6]. Loss of photosynthetic activity is postponed by glycine betaines which can inhibit chlorophyll degradation during storage in remote chloroplasts [29]. It was also reported that seaweed extracts can promote early flowering and fruiting in a variety of crops [5]. Since crop yield is linked with flower number at the mature stage, seaweed extracts can promote flowering by influencing rapid plant growth and switching from vegetative to reproductive growth [1,30]. This increase in reproductive growth was seen in both tomato and sweet pepper and was also reported in several studies [5,6]. Hormonal substances present in these extracts have been shown to stimulate an increase in endogenous phytohormones comprising of auxin, IAA cytokinins and gibberellic acid, which can cause improvement in plant growth and yield of treated plants [8]. Isopentenyltransferases (IPT) are the first components involved in cytokinin biosynthesis [31] and it has been shown that seaweed extracts lead to increases in transcripts of genes involved in their production. Seaweed extracts also lead to significant upregulations of key gene transcripts involved in gibberellin and auxin synthesis in tomato and sweet pepper plants. This upregulation of hormonal gene transcripts may have occurred due to AN application which influenced endogenous synthesis of hormones via the activation of respective genes involved in their production [24,32]. Furthermore, the extract is reported to contain minimum levels of auxin, cytokinins, gibberellins and their analogues. These hormones may work in tandem to promote efficient growth directly or via various crosstalk mechanisms. Additionally, the macronutrients and micronutrients present in the extracts may also account for some of these growth benefits [14].

Generally, the usage of *A*. *nodosum* extract has resulted in significant increases in crop yields and reduction in plant disease levels. The extract seemed to perform more effectively in the tropical environment compared to temperate and subtropical zones as noted from existing reports. Greater activity was observed when *A*. *nodosum* extract was combined into an integrated cropping system wherein sprays were alternated with very minimum level of fungicides. The use of organic seaweed extract inputs in crop production presents a valuable and significant cost saving practice compared to the chemical inputs particularly for small and medium farm holdings. The organic nature of the extract also makes it very appealing to both farmers, consumers and government bodies towards its inclusion in sustainable agriculture.

## Supporting information

S1 Table This is the S1 Table with the Oligonucleotide primers used in the study. (PDF)

S1 Dataset This is the S1 Dataset with the raw datasets obtained from the study. (XLSX)
