# Supplementary material for: Biostimulatory activities of Ascophyllum nodosum extract in tomato and sweet pepper crops in a tropical environment
Source: PLoS One. 2019 May 14;14(5):e0216710. doi: 10.1371/journal.pone.0216710 (PMC6516672; doi:10.1371/journal.pone.0216710)
Supplement: S1 Table — (PDF) [file pone.0216710.s001.pdf]

Table 1: Oligonucleotide primers used in the study

Organism	Gene	Sequence	Function	Amplicon size (bp)	Reference
A. solani	*SP-sol-1658*	F-GTAGAGTATGTTGAATACTCTAACCAG ACAA	Species specific primer for *A. solani*	437	[1]
		R-ATGTTAAGAATTTGTCCTGAACAGTTT			
X. campestris pv. *vesicatoria*	*XV1*	F- CAGTCCTCCAGCACCGAAC	Species specific primer for *X. c* pv.*vesicatoria*	275	[2]
		R- TCTCGTCGCGGAAGTACTCA			
Tomato-*Solanum lycopersicum*	*β-actin*	F-CTCGAGCAGTGTTTCCCAGT	House-keeping gene	247	
		R-GGTGCCTCAGTCAGGAGAAC			
	PR-1a	F- TCCTCCATTTTCGTTGCTTGT	*PR-1*, SA-inducible defence gene	281	
		R- TCGTCCCACATCTTCACAGC			
	PIN II	F-ACGACGTGTTGCACTGGTTA	*Pin-II*, JA-inducible gene	198	
		R-GCAACCCTCTCCTGCACTAC			
	ETR-1	F-TGGTGTCATGTCCTTGCTGG	Ethylene response gene	70	
		R-CTGAGTAGCGTGGCTGTGAT			
	GA2ox 1-Sl	F-GCCATGCTCAGAGATTGAACGATTG	Gibberellin 2 oxidase-gibberellin biosynthesis	530	[3]
		R-CCCACAATGAGCATCTTGACAACC			
	IAA-Sl	F-CTCCAGGGTGATTTCTGT	Indole-3-acetic acid-amido synthetase	196	[4]
		R-TTCTTTGGTCCACTGTCT			
	IPT-Sl	F-ACTCTCTATCGACTTAGCCACTCAA	Cytokinin biosynthesis-adenylate isopentenyltransferase)	144	[5]
		R-ATTACCCCTAGGCAATGATGTGGT			

Pepper-*Capsicum annuum*	*GA2ox 1-Ca*	F-GCGATACATTTATGGCGCTTTCA	Gibberellin 2 oxidase-gibberellin biosynthesis	184	[6]
		R-CATGTGAGGTCGGGATACATTCG			
	IAA-Ca	F-ACCCGCGTGGTCGAGTACACTAGTTATGC	Indole-3-acetic acid-amido synthetase	392	[7]
		R-CGACGTCGTTCAGGTGTCCATTGAGGC			
	IPT-Ca	F-CCCTCCGTACCCATTTTCCCA	Cytokinin biosynthesis-adenylate isopentenyl transferase	239	Self-designed
		R- ATTTTCCAGCCCCGGTCACT			
	β-actin	F-AGGGATGGGTCAAAAGGATGC	Actin, House-keeping gene	290	[6]
		R-GAGACAACACCGCCTGAATAGC			
	CaPR-1a	F-CTGGTGCCGTGAAGATGTGGGT	*PR-1*, SA-inducible defence gene	157	[8]
		R-TACCACCCATTGTTGCACCGAA			
	CaPin-11	F-ATGGCTGTTCCCAAAGAAG	*Pin-II*, JA-inducible gene	292	[9]
		R-CTGTTCATGCTTTTACTTTTC			
	CaETR-1	F-CCTAAACGCCTCTTTGGTCT	*Etr-1*, Ethylene response gene	348	[10]
		R-TGCTATTGGCTACTCTTCTG			

References

1. Vandecasteele M, Landschoot S, Carrette J, Verwaeren J, Höfte M, Audenaert K, et al. Species prevalence and disease progression studies demonstrate a seasonal shift in the *Alternaria* population composition on potato. Plant Pathol. 2018; 67: 327–336.

2. Ramkissoon A, Ramsubhag A, Jayaraman J. Phytoelicitor activity of three Caribbean seaweed species on suppression of pathogenic infections in tomato plants. J Appl Phycol. 2017; 34: 123–129.

3. Rayorath P, Khan W, Palanisamy R, Mackinnon SL, Stefanova R, Hankins SD, et al. Extracts of the brown seaweed *Ascophyllum nodosum* induce gibberellic acid (*GA3*)-independent amylase activity in barley. J Plant Growth Regul. 2008; 32: 123–128.

4. Lee Y, Kim YC, Kim SY, Lee IJ, Choi D, Paek KH, et al. A novel gibberellin 2-oxidase gene *CaGA2ox1* in pepper is specifically induced by incompatible plant pathogens. Plant Biotechnol Rep. 2012; 25: 111-113.

5. Zhang Y, Li Z, Tu Y, Cheng W, Yang Y. Tomato (*Solanum lycopersicum*) *SlIPT4*, encoding an isopentenyltransferase, is involved in leaf senescence and lycopene biosynthesis during fruit ripening. BMC Plant Biol. 2018; 28: 233-240.

6. Lai Y, Xu B, He L, Lin M, Cao L, Mou S, et al. Differential gene expression in pepper (*Capsicum annuum*) exposed to UV-B. Indian J Exp Biol. 2011; 6: 134–145.

7. Liao D, Chen X, Chen A, Wang H, Liu J, Liu J, et al. The characterization of Six auxin-induced tomato GH3 genes uncovers a member, SlGH3.4, strongly responsive to arbuscular mycorrhizal symbiosis. Plant Cell Physiol. 2015; 34: 123-144.

8. Zhang YL, Li DW, Gong ZH, Wang JE, Yin YX, Ji JJ. Genetic determinants of the defense response of resistant and susceptible pepper (*Capsicum annuum*) cultivars infected with *Phytophthora capsici* (Oomycetes; *Pythiaceae*). Genet Mol Res. 2013; 20: 100-110.

9. Mishra M, Mahajan N, Tamhane VA, Kulkarni MJ, Baldwin IT, Gupta VS, et al. Stress inducible proteinase inhibitor diversity in *Capsicum annuum*. BMC Plant Biol. 2012; 12: 54–66.

10. Onate-Sanchez L, Singh KB. Identification of Arabidopsis Ethylene-Responsive Element Binding Factors with Distinct Induction Kinetics after Pathogen Infection. Plant Physiol. 2002; 7: 45–66.
